# Surface Defects and Crystal Growth of Apremilast Benzoic Acid Cocrystals

**DOI:** 10.1021/acs.oprd.4c00480

**Published:** 2025-03-19

**Authors:** Jan Jirát, Vít Zvoníček, Luděk Ridvan, Miroslav Šoóš

**Affiliations:** a Department of Chemical Engineering, University of Chemistry and Technology, Technicka 3, Prague 6,Dejvice 166 28, Czech Republic; b Zentiva, k.s., U kabelovny 130, Prague 10 10237, Czech Republic

**Keywords:** apremilast benzoic acid cocrystal, crystallization, two-phase growth of the crystals, crystal dissolution

## Abstract

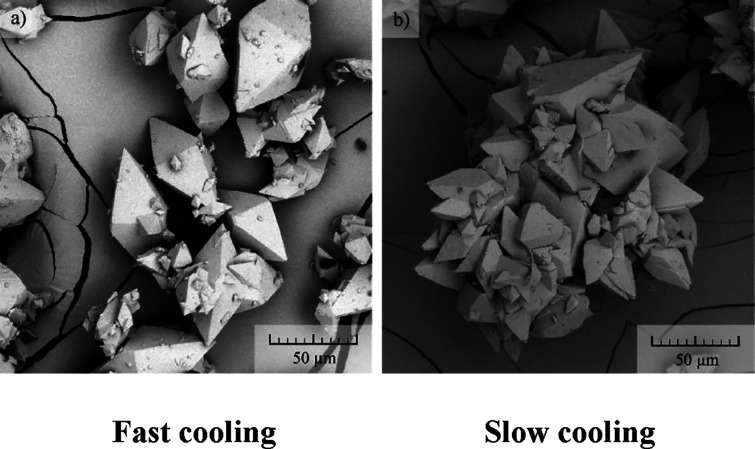

A cocrystallization process of the active pharmaceutical ingredient apremilast with benzoic acid is explored in this work. The aim of the study is to adjust operating conditions during the crystallization to purposefully tune the dissolution properties of the final product. Understanding the cocrystallization is key to obtaining a consistent, high-quality product, as well as tuning other properties such as powder flowability or dissolution properties. It was discovered early in development that the studied cocrystallization process does not follow the common rules of crystallization. Better crystals were obtained at faster cooling rates and worse crystals at slower cooling rates. Interestingly, this can be explained by crystal collisions and a two-phase growth of the crystals. Standard operating conditions were further tested, resulting in different shapes and sizes of the product. Different types of produced crystals were tested in a dissolution apparatus and provided significantly modified dissolution profiles.

## 1 Introduction

The pharmaceutical industry is challenged with the task of increasing poorly water-soluble products in the last couple of decades.^1−3^ The low solubility translates directly to low bioavailability,^4,5^ which is undesirable, especially for the case of orally administered drugs, which is the most common way of administration. This industry-wide problem is expected to increase even further from approximately 40%^6^ of the drug being poorly soluble in the current marked at 70–90% in the future.^7^ Alternative solid forms of the active pharmaceutical ingredient (API), such as cocrystals, salts, and solvates, appear as one of the potential solutions for this problem.^8^ These solid forms offer unique physicochemical properties and diversify the solid-state landscape of the API.^2,8^

Ongoing advances in the field of crystal engineering make the design and subsequent preparation of multicomponent forms, including polymorphic forms, more and more affordable. This allows us to identify and select the most suitable solid form for further drug product development. Two basic approaches are used during the screening process, solution-based and nonsolvent-based methods. The solution-based methods are more established due to solvent diversity that can be utilized and include solution crystallization, antisolvent crystallization, vapor diffusion, and slurry-based screenings to name a few.^9^ Nonsolvent methods are recently gaining momentum in the screening part of the drug development, particularly the mechanochemical approach or crystallization from melt.^10,11^ Nonsolvent methods have the added benefit of being environmentally friendly and also remove the interaction of the solvent with the API, which can be undesired. However, the final step in any API production is usually crystallization (more than 90% of APIs are delivered in crystalline form^12^); therefore, the solution-based screening methods provide valuable insight into the production challenges. The final crystallization is an important step in the API production as it impacts several key properties of the selected candidate: purity of the material, particle size, shape, and crystal defect rate.^13^ Small adjustments in the operating conditions, such as supersaturation, cooling rate, stirring speed, or seeding, can impact the properties of the crystals substantially, both in bioavailability and subsequent processability of the material. Therefore, it is vital to properly understand the crystallization of active pharmaceutical ingredients.

In the case of multicomponent forms, such as cocrystals or salts, the crystallization process becomes even more complicated. Crystallization of multicomponent forms always bears the risk of crystallizing only a single compound or unwanted mixtures of a single compound and the multicomponent form resulting in a devaluated product.^14^ Ternary phase diagrams are often used to minimize these risks and experimental efforts.^15,16^ We have previously discovered a premilast benzoic acid cocrystal and constructed its ternary phase diagram for several solvents. The apremilast benzoic acid cocrystal provides significant improvements compared to the polymorph of apremilast used in the original drug product.^17^ In this study, we further explore this system toward understanding the impact of operating conditions during crystallization with the intention to tune the cocrystal properties. The explored conditions were the cooling rate, stirring speed, seeding policy, and impeller type. Tuned properties of the final product were the shape and size of the crystals. Various batches of products with modified properties were tested in the dissolution apparatus to confirm the desired modification of the final product dissolution properties.

## 2 Experimental Section

### 2.1 Materials

Methyl ethyl ketone was purchased from PENTA (Czech Republic, Chrudim) and used as received without further purification. Benzoic acid (99%) was purchased from Alfa Aesar (Germany, Karlsruhe) and was milled in a vibratory mill (Retsch MM 200, Retsch, Germany) to decrease the particle size. Metal milling jars and balls (3 mm diameter) were used at a frequency of 25 Hz. Apremilast, used for the treatment of psoriasis and psoriatic arthritis,^18^ was synthesized and kindly provided by Zentiva k.s. (Czech Republic). In particular, its form II was used in all the experiments mentioned below as a starting material. The impact of process parameters on the crystallization process was investigated in an EasyMax system 102 with a vessel volume of 100 mL with a filling volume equal to 50 mL. The diameter of the bottom dish vessel was 50 mm, and the height of the liquid level was 27 mm. The diameter of the four-pitched blade impeller was equal to 25 mm with a blade of 10 × 7.7 (radial dimension) mm. The down-pumping impeller was located 10 mm from the vessel bottom. The concentration of apremilast during crystallization experiments was 100 mg/mL.

### 2.2 Methods

#### 2.2.1 X-ray Powder Diffraction (XRPD)

The diffraction patterns were collected with a powder diffractometer device X’PERT PRO MPD PANalytical: X-ray beam Cu Kα (λ = 1.542 Å), measured range: 2–40° 2Θ, excitation voltage: 45 kV, anodic current: 40 mA, step size: 0.01° 2Θ, remaining at a step for 0.05 s. The measurement was performed on a flat sample with an area/thickness ratio of 10/0.5 mm. The 0.02 rad Soller slits, 10 mm mask, and 1/4° fixed antiscattering slits were used to correct the primary beam. The irradiated area of the sample was 10 mm; programmable divergent slits were used. The 0.02 rad Soller slits and 5.0 mm antiscattering slits were used to correct the secondary beam. HighScore Plus software was used to process the diffraction patterns.

#### 2.2.2 Raman Spectroscopy

Samples for Raman spectroscopy were measured in HPLC glass vials in a spectrometer device FT-Raman RFS100/S, with a germanium detector (Bruker Optics, Germany). The wavelength of the Nd:YAG laser was 1064 nm. The measuring range was from 4000 to 200 cm^–1^, with a spectral resolution of 4.0 cm^–1^. Data were obtained at either 64 or 128 accumulations of the measured spectra. The software OMNIC and OPUS were used to process the obtained Raman spectra.

#### 2.2.3 Scanning Electron Microscopy (SEM)

The shape and size of the particles were determined by scanning electron microscopy. All samples were gold-coated using a Q150R ES Plus device (Quorum Technologies, United Kingdom). A Tescan Mira/LMU device was used for the SEM analysis. The images were taken by the detector of a backscattered electron (BSE), and the acceleration voltage was set to 5 kV.

#### 2.2.4 Dissolution Measurement

Dissolution profiles were measured in pH = 2 water at 150 rpm (RPM). The dissolved amount of the sample was measured over 60 min using a Cary 60 UV–vis spectrophotometer (Agilent Technologies, USA). A wavelength of 340 nm was used to determine the dissolved amount of the API. All experiments were performed in 500 mL of dissolution media into which we added 7.5 mg of cocrystals, which corresponds to the maximum amount of API equal to 0.013 mg/mL.

#### 2.2.5 Focused Beam Reflectance Measurement

A ParticleTrack G400 probe (Mettler Toledo, Switzerland) with a probe diameter of 9.5 mm was used to obtain chord length distribution (CLD) employing a scanning speed of 2 m/s. The macro chord selection method was used for evaluation of the chord length distribution. The measurement was performed over the entire duration of the experiment. Chord length distribution was evaluated every 5 s.

## 3 Results and Discussion

Operating conditions were explored during the cocrystallization process to provide final tuning of the cocrystal and ensure the consistent quality of the product. Cocrystallization experiments were performed in the crystallization systems EasyMax (Mettler Toledo) and Crystalline (Technobis Crystallization Systems).

**Figure 1:**
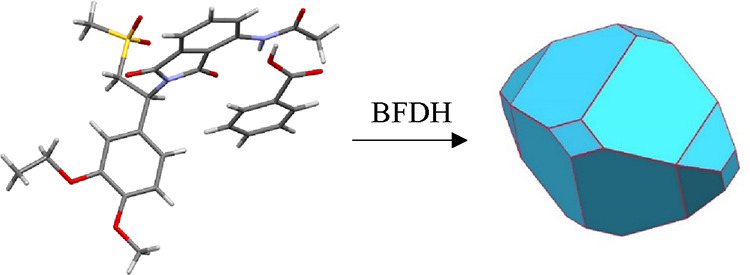
Crystal shape was calculated from the solved structure by the BFDH model.

The crystal shape was predicted before the start of the crystallization experiments (see Figure 1). The shape prediction was calculated via the BFDH model^19−21^ in software Mercury^22^ from the crystal structure of the cocrystal.

### 3.1 Two-Phase Crystal Growth

**Figure 2:**
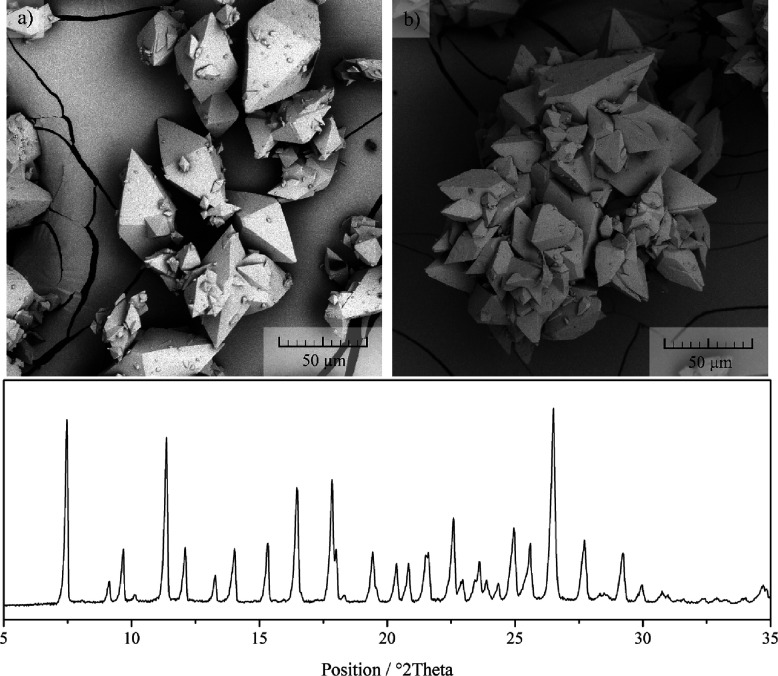
Comparison of the two distinct habit types: (a) single crystals and (b) crystal clusters, together with the XRPD diffractogram.

Nonseeded experiments were performed first to obtain information about the crystallization process itself. The general procedure of the initial experiments was to heat up the solution of apremilast, benzoic acid, and methyl ethyl ketone to 70 °C, keep it at elevated temperature for 30 min until all materials dissolved, and cool it down to −10 °C. The product from initial experiments was filtered, dried (24 h, 40 °C, 100 mbar), and characterized by Raman spectroscopy and XRPD confirming cocrystal formation in all cases. Consequently, the detailed shape of prepared crystals and their surface morphology was characterized by SEM. Interestingly, the formation of two different habit types was observed: single crystals (see Figure 2a) and their clusters (see Figure 2b).

When considering simplicity and limitations of the BFDH model, formed single crystals match the crystal prediction well. A small difference can be seen toward the tip of the bipyramid, where the flat surface does not evolve in real crystals. It is important to emphasize that crystal clusters (Figure 2b) are formed from the single crystals (Figure 2a). Please note that the difference between them is not in the habit of the crystal but in the overall habit of the product.

**Figure 3:**
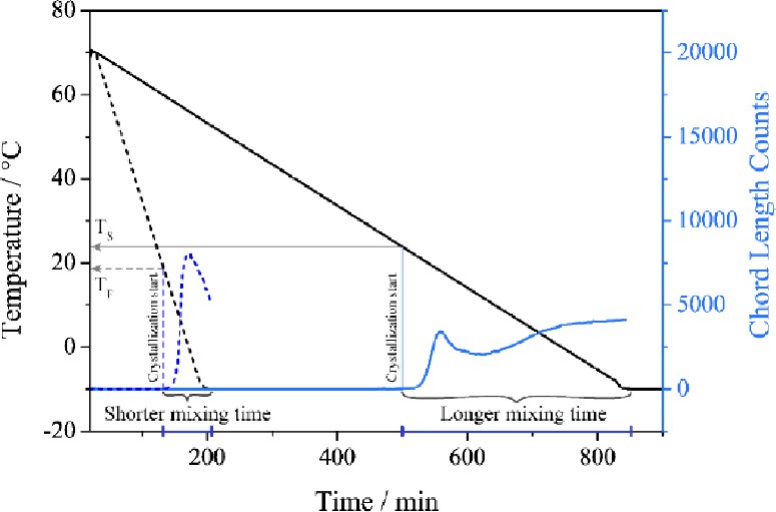
Faster and slower cooling rate and chord length count for both experiments. The chord length was measured over the range from 0 to 1000 μm.

**Figure 4:**
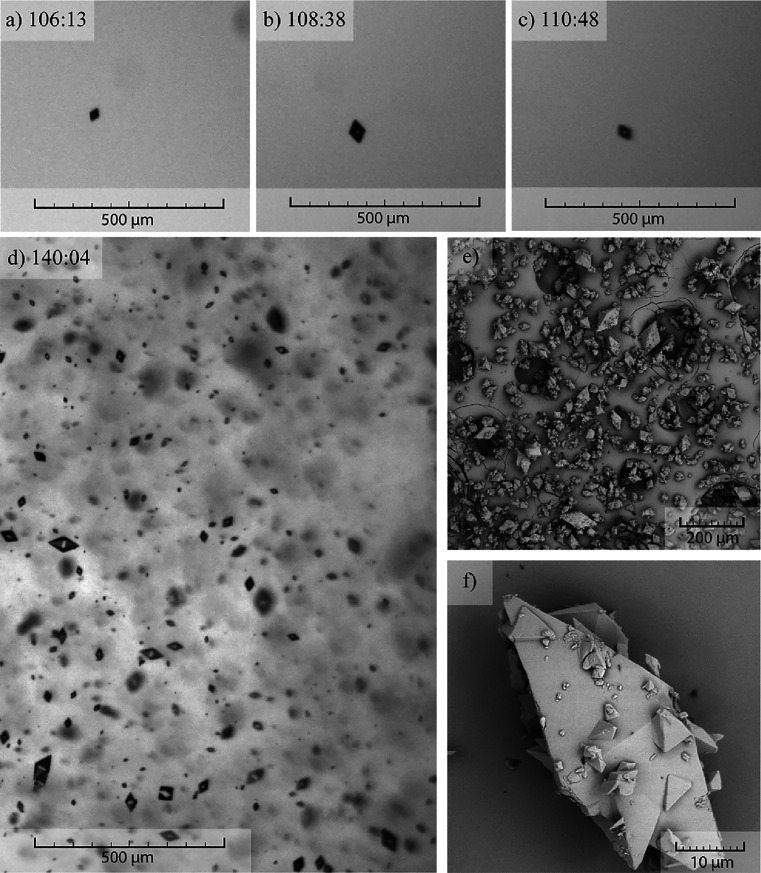
(a–c) Single crystals formed after the crystallization onset. (d) Reactor filled with crystals. (e, f) SEM pictures of the crystals from the same experiment. Cooling rate, 0.5 °C/min.

**Figure 5:**
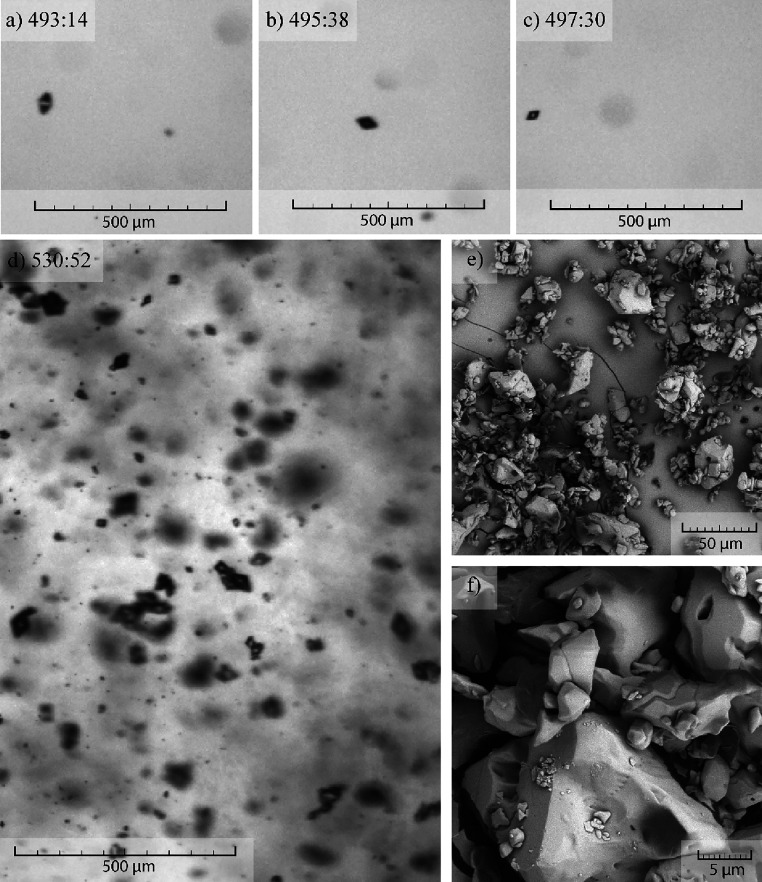
(a–c) Single crystals formed after the crystallization onset. (d) Reactor filled with crystals. (e, f) SEM pictures of the crystals from the same experiment. Cooling rate, 0.1 °C/min.

Initial experiments leading to formation of crystals in Figure 2 were performed at equal operating conditions except for cooling rate. In the case of crystal clusters (Figure 2b), the cooling rate was slower than in the case of single crystals (Figure 2a). This phenomenon was not expected since the growth of better crystals, with less crystal flaws, is commonly proportional to slower cooling rates during crystallization.^23−27^ Thus, obtaining better, well-separated crystals at a faster cooling rate is quite a unique phenomenon. The habits of individual crystals are similar in both crystal clusters and single crystals. However, the overall habits of the product is different. Since the similarity of the crystal habit is striking, it was assumed that nucleation is equal in both product types, and the difference is formed during the crystal growth period. This phenomenon was further studied by combining the crystallization experiment in the EasyMax system to measure crystal growth as well as the onset of the crystallization using the ParticleTrack probe and system Crystalline, which is composed of an optical system to monitor specifically the growth of the crystals. Both crystallization systems work with reactors of different volumes and stirrers (8 mL reactor with a hook stirrer for Crystalline and 50 mL reactor with a pitched four-blade stirrer for EasyMax). Therefore, the hydrodynamics in the reactors are different, but each system provides valuable optical and harmonic length distribution (CLD) data to understand system evolution during cooling crystallization. Experiments proceeded without seeding under equal conditions with the exception of the cooling rate. The procedure of the experiments consisted of heating to 70 °C until complete dissolution of the solid phase and cooling to −10 °C. Tested cooling rates were 0.5 °C/min (faster) and 0.1 °C/min (slower) to confirm that the cooling rate is the crucial parameter that leads to the formation of either crystal clusters or separated crystals. The obtained temperature profile with the CLD signal from EasyMax and ParticleTrack probe is presented in Figure 3, while optical images of the formed crystals as a function of time are shown in Figure 4 (0.5 °C/min) and in Figure 5 (0.1 °C/min).

The ParticleTrack probe was used to detect the onset of crystallization during experiments. Figure 3 shows crystallization onsets at different temperatures at different cooling rates. At a cooling rate of 0.1 °C/min, the crystallization started at approximately 24 °C, whereas at 0.5 °C/min, it started at approximately 18 °C. The trend of the crystallization onset at higher temperature using various cooling rates was confirmed by an additional experiment (see Figures S3 and S4). Consequently, the remaining time between the onset of crystallization and the final temperature (end of the experiment) is longer for slower cooling speeds. The optical data from system Crystalline were compared with the ParticleTrack data to provide further insight into the crystallization process.

**Figure 6:**
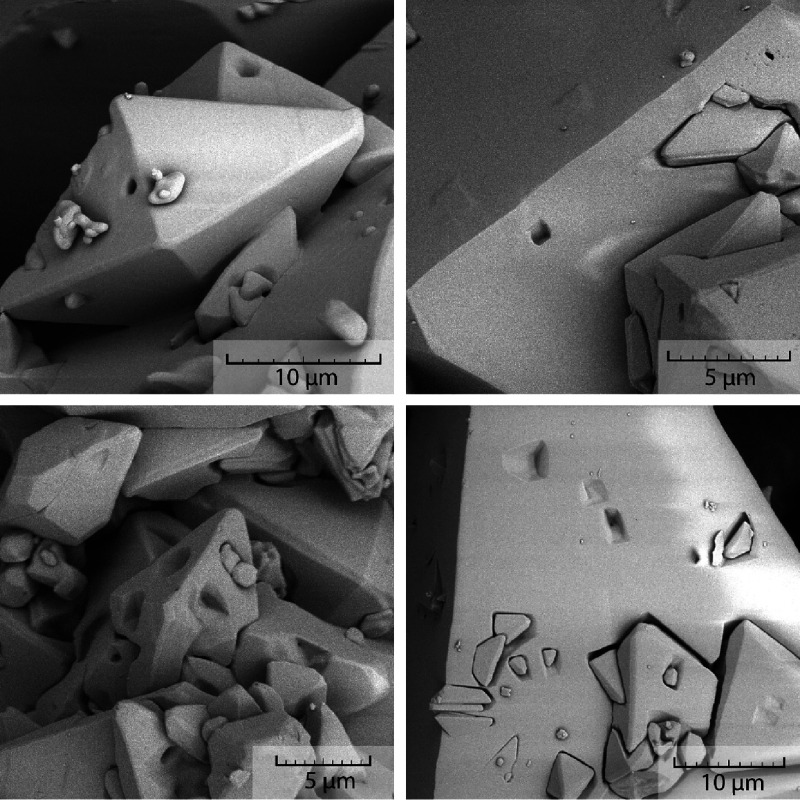
Close-up examination of crystals by SEM obtained for 150 rpm, amount of API during crystallization equal to 100 mg/mL, no seeding, and cooling rates of 0.8 °C/min (top row) and 0.1 °C/min (bottom row).

Optical monitoring of experiments throughout the entire duration provided valuable data. For the case of slower cooling rate (0.1 °C/min), crystallization onsets at higher temperature (approximately 25 °C). This means that crystallization starts from a less supersaturated solution (*S* = 4) compared with the faster cooling rate, in which case the crystallization starts around at 18 °C and higher supersaturation (*S* = 8). It is important to note that it is possible to see similar, well-separated single crystals at the start of the crystallization in each experiment (Figures 4a–c and 5a–c). This confirms that initially, the apremilast cocrystals are crystallizing as individual crystals, and formation of crystal clusters happens later in the process as a result of crystal–crystal collisions. In fact, as the crystallization continues, the concentration of solid particles inside the reactor increases and it is possible to observe differences in the overall habit of the product (Figures 4d and 5d). SEM pictures present in Figures 4e,f and 5e,f show substantial difference between the habit of the produced crystals. The experiment with a faster cooling rate results in single crystals scarcely covered with small crystals (see Figure 4e,f). On the other hand, crystallization with a slower cooling rate results in a more cluster-like product that is however heavily damaged by the collisions between the crystals themselves and the stirrer (see Figure 5e,f). It is striking that crystals from the slow cooling experiment suffered significantly from more damage compared to a faster cooling rate. Crystals from the crystallization experiments were closely examined using SEM to find a similar level of damage exerted onto the crystals (see Figure 6).

**Figure 7:**
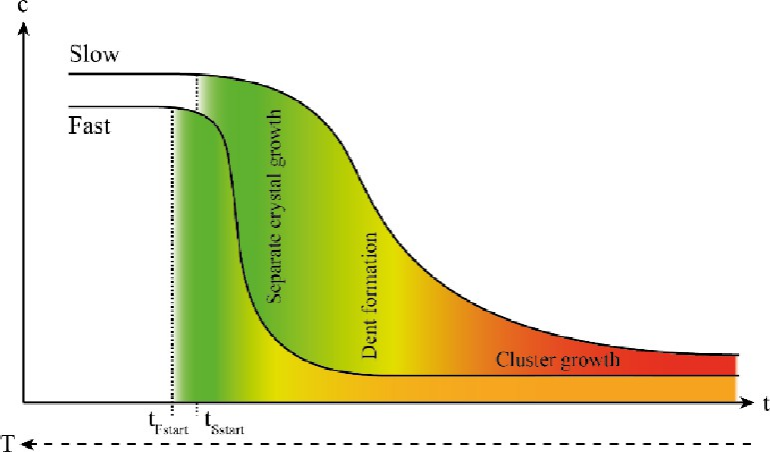
Schematic representation of crystal growth during both cooling rates.

The close-up examination of the crystal immediately reveals damage on their surface in the form of indents. These indents are most probably resulting from the collisions between the crystals and possible between crystals and the impeller. However, the size and shape of the indents suggest formation by collision of the flat surface of one crystal with the top of the bipyramidal shape of another crystal. Figure 6 also reveals that new crystals can grow from the indent and thus connect with the original crystal. Repetitive growth of new crystals from the surface indents leads to the formation of the crystal cluster habit. This mechanism is also supported by the fact that the crystal clusters are very durable as opposed to clusters formed by aggregation of separate particles. These clusters do not get separated or damaged by any subsequent operations after the crystallization (i.e., filtration, drying, transport between different containers, analysis, etc.). This mechanism of cluster growth would explain why using a slower cooling rate results in clusters, whereas a faster cooling rate results in separated crystals. While using a slower cooling rate, the crystallization starts at a higher temperature (in the case shown in Figure 5 at approximately 25 °C). After the onset of crystallization, a significantly longer time remains until the end of the process (approximately 350 min) due to the slower cooling and higher crystallization onset temperature. During the remaining time, the amount of solid phase in the reactor increases and therefore the probability of the collisions rises. This process keeps repeating and generates indents that work as a site for growth of connected crystals resulting in the product cluster habit change at the end of the crystallization. On the other hand, during the faster cooling rate, the crystal collisions are significantly reduced, i.e., the time between the onset and termination of the crystallization is shorter (in the case shown in Figure 4, it is approximately 60 min). Hints of the crystal cluster habit are present in the case of a faster cooling rate as well (Figure 4f), but since the supersaturation is already low at the time of indent formation, crystal clusters cannot fully grow. Overall, the shorter time of cooling and lower temperature of the crystallization onset are both beneficial for formation of separated crystals as opposed to clusters. Schematic representations of the crystallization process for both cooling rates are displayed in Figure 7.

Please note that the time axis is accurate for different cooling rates, but the temperature axis is just schematic as both cooling rates start and end at equal temperatures. The growth of the product can be separated into two phases. The first phase, with high supersaturation, includes separated crystals. In the second phase, with lower supersaturation, clusters grow from the surface dents created by the collisions instead of the separated crystals that would require new nuclei to form. The two phases cannot be strictly separated and are likely overlapping during the dent formation. Thus, the difference in the resulting habit of the product is not driven by supersaturation levels in the traditional way but by the two-phase growth of the product.

### 3.2 Operating Conditions

Based on the above-mentioned change of the crystal habit, the set of operating conditions was tested to tune the product properties. These included mixing intensity, type of stirrer, and seeding policy.

#### 3.2.1 Mixing Speed and Stirrer Type

**Figure 8:**
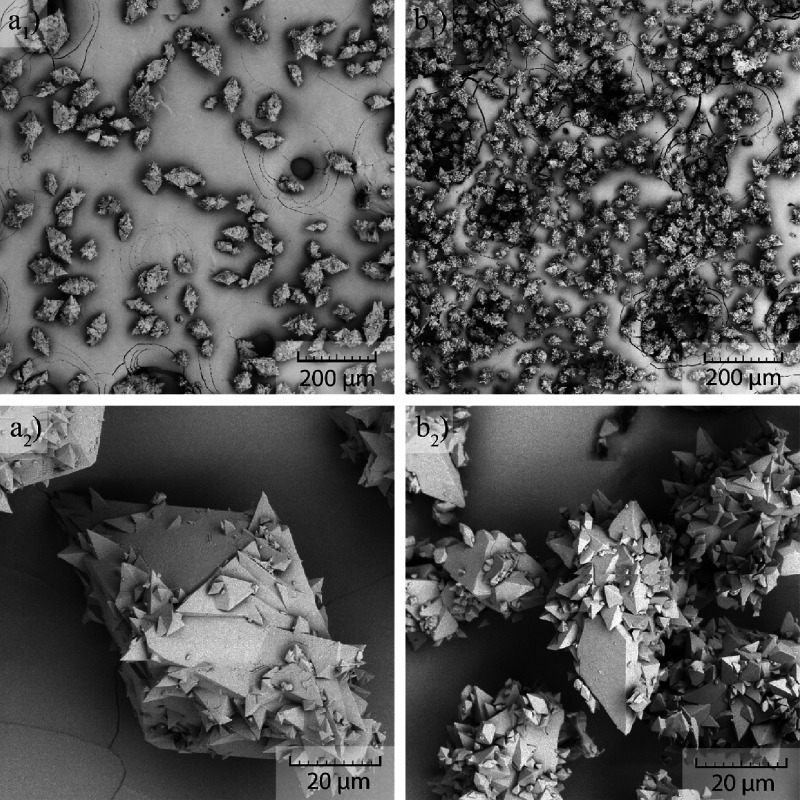
SEM image of the product at (a) 150 and (b) 500 rpm.

The impact of the mixing speed (stirrer RPM) was explored in two equal experiments (similar to experiments described above), once using 150 rpm and second time using 500 rpm using a four-blade pitched stirrer. The lower stirring speed results in overall bigger crystals with a size of approximately 80 μm from top to top of the bipyramid (see Figure 8a_1_,a_2_). The crystals obtained using higher stirring speed are of a size of approximately 45 μm (see Figure 8b_1_,b_2_). It is interesting that crystals produced at 150 rpm are closer to the separated crystal habit, whereas at 500 rpm, the product is more cluster-like. This further points out the mechanism of dent formation and two-phase crystal growth.

Since the cooling rate and supersaturation were equal in these experiments, the faster stirring speed, i.e., 500 rpm, increased the intensity and probability of crystal collisions. Therefore, the surface dents formed earlier in the experiment, at higher supersaturation, and the crystal clusters could grow. For the case of slower mixing, the supersaturation is already low at the time of indent formation. This results in clusters that are not nearly as developed as for the faster stirring speed.

#### 3.2.2 Seeding Policy

**Figure 9:**
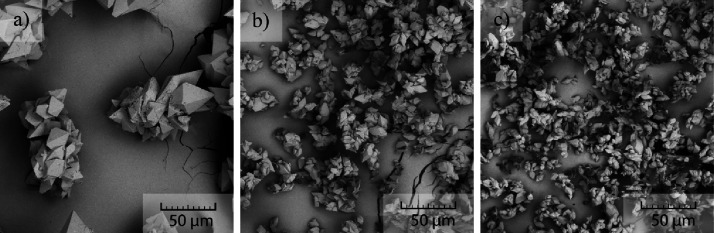
Resulting product with various amounts of seeding used: (a) 0.4%, (b) 5.2%, and (c) 12%.

Seeding consisted of partially clustered particles of small size (approximately 5 μm). Three identical experiments were conducted with different amounts of seeding. The masses of the seeding were 10, 130, and 300 mg, which are 0.4, 5.2, and 12% of the apremilast and benzoic acid mass dissolved in the solution, respectively. The impact of the seeding on the final product is displayed in Figure 9.

As the amount of seeding increases, the size of the final product decreases. This is due to more growing sites being introduced into the system. Equal supersaturation was used in the experiments; therefore, with more seeds, the resulting particles are smaller, and with less seeds, the product can grow larger. The seeding amount proved to significantly impact the size of the product. In Figure 9, the size of the product varies from approximately 15 to 100 μm.

#### 3.2.3 Stirrer Type

**Figure 10:**
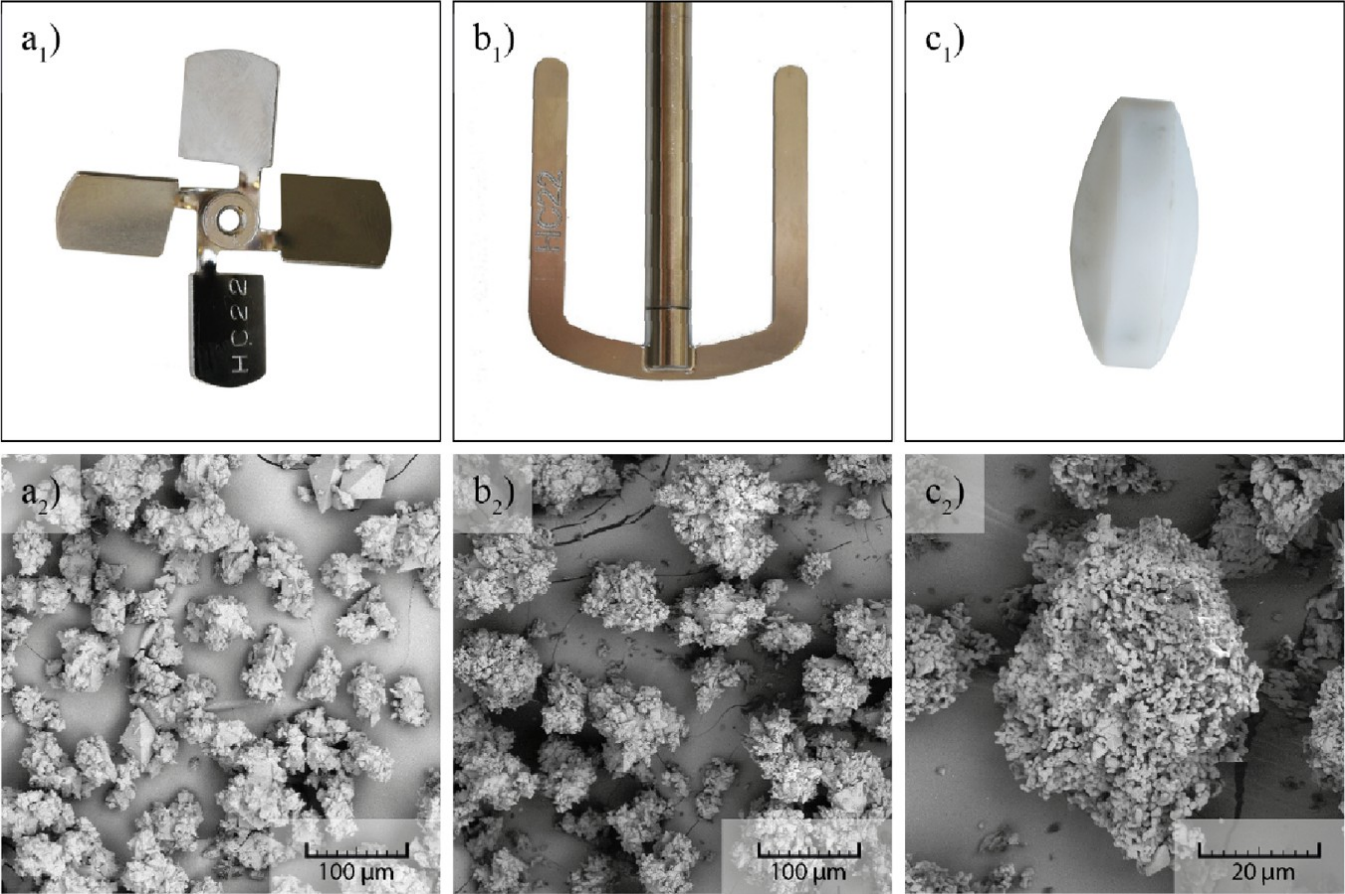
Crystals produced using different types of stirrers: (a) pitched four-blade overhead stirrer, (b) anchor overhead stirrer, and (c) pill-shaped magnetic stirrer.

Different types of stirrers were tested to determine their impact on the final product. Two types of overhead stirrers are used as well as a magnetic stirrer. The overhead stirrers were a pitched four-blade stirrer (Figure 10a) and anchor stirrer (Figure 10b). The magnetic stirrer used was a pill-shaped stirrer (Figure 10c). The different stirrers produce different hydrodynamics within the reactor, and the magnetic stirrer introduces also friction between the stirrer and the glass of the reactor.

As can be seen from Figure 10, the difference between the overhead stirrers was negligible. However, the magnetic stirrer produced a completely different habit of the final crystals. It consists of very small crystals (approximately 2 μm) connected into bigger clusters. The crystallization is induced by the friction of the magnetic stirrer and the glass wall of the reactor. The friction produces a large number of seeds resulting in significantly smaller crystals. At the same time, the magnetic stirrer grounds already formed crystals against the glass wall, further reducing their size.

### 3.3 Dissolution Measurements

Dissolution measurements were performed to reveal whether the adjustments of the crystallization process bring desired changes in dissolution profiles and potentially subsequent bioavailability. Products of different overall habits and sizes were tested during dissolution. The tested crystals were used after filtration and drying, but no further process steps were applied (milling, sieving, micronization, etc.). Testing crystals without additional process steps allows for a direct correlation of the dissolution profiles with the produced crystals.

**Figure 11:**
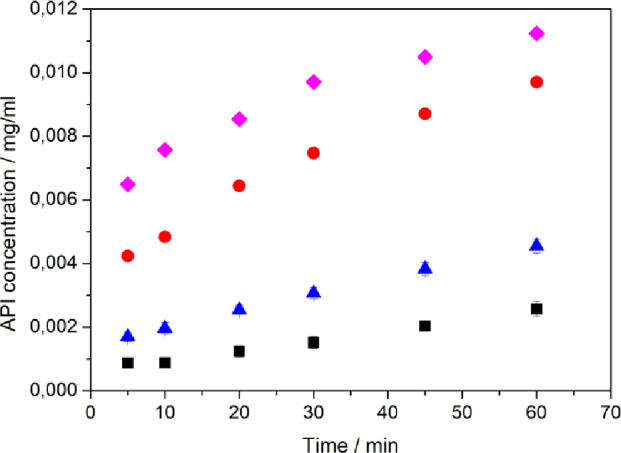
Dissolution profiles for products of different shapes and sizes. Black square, big clusters, size, 125 ± 25 μm; blue triangle, big bipyramids, size, 85 ± 15 μm; red circle, small clusters, size, 40 ± 10 μm; magenta diamond, small bipyramids, size, 4 ± 1.5 μm. All experiments were performed in 500 mL of dissolution media where we added 7.5 mg of cocrystals, which corresponds to the maximum amount of dissolved API equal to 0.013 mg/mL. Corresponding SEM images are presented in Figure S5.

Four different product batches were tested in the dissolution apparatus. Small and large particles were characterized by crystal cluster habit and separated crystal habit. The large sizes of crystal clusters and separate crystals were 125 ± 25 and 85 ± 15 μm, respectively. Sizes of small and large separated crystals were 40 ± 10 and 4 ± 1.5 μm, respectively. Figure 11 clearly shows the differences in dissolution among the four tested batches. Smaller size of the product results in faster dissolution for both crystal clusters and separated crystals. It is interesting to note that already at the fifth minute mark, there is a significant increase in the concentration. The offset is higher for smaller product sizes. This quick concentration increase early in the experiment might be caused by rapid dissolution of very small crystals that are produced during the crystallization as well. Attrition of crystals between crystallization and dissolution experiments might also contribute to the rapid concentration increase at the start of the dissolution measurement. Reproducibility of the measurement was tested for two product batches (error bars are plotted), and similar deviation was assumed for the rest of the dissolution experiments. Overall, the adjustments of the crystallization process, and in turn, the shape and size of the final product, provide an additional modification of the dissolution profiles without the need for further process steps.

## 4 Conclusions

A cocrystallization process for a pharmaceutical cocrystal of apremilast and benzoic acid was successfully developed. The initial production revealed that the formation of two different types of product habits is possible: separate crystals (bipyramidal shape) and crystal clusters. Interestingly, crystal clusters formed during the use of a slower cooling rate and separate crystals formed at a higher cooling rate. This phenomenon is in contradiction with the common rules of crystallization since the defects of the crystal lattice appear more readily at higher supersaturations caused by faster cooling rates. However, in this case, it was discovered that collisions between crystals cause significant damage to the crystal surface. Observed indents on the crystal surface act as nucleation centers for the growth of subsequent crystals. The repetition of this process leads to the formation of crystal clusters. This results in a two-phase growth mechanism (growth–collision–growth) during the crystallization. In experiments with slower cooling rates, the time of the experiment is longer; therefore, the time for collisions and growth of clusters also increases. Correct settings of key operating conditions, such as cooling rate, mixing speed, and type of impeller, need to be established to purposefully lead the two-phase growth crystallization toward the formation of the desired product. It was discovered that dissolution profiles can be substantially modified if the crystallization process is performed properly. This saves both effort and expenses required to incorporate additional process steps to further tune the product properties.

## Supporting Information Available

Raman spectra and XRDP patterns measured for sample batches, time evolution of temperature, chord length counts, and in situ optical images of crystals for the three cooling rates used in the study (PDF)
