# Supplementary material for: Surface Defects and Crystal Growth of Apremilast Benzoic Acid Cocrystals
Source: Org Process Res Dev. 2025 Mar 19;29(4):1067–75. doi: 10.1021/acs.oprd.4c00480 (PMC12012881; doi:10.1021/acs.oprd.4c00480)
Supplement: Supplementary file 1 — op4c00480_si_001.pdf [file op4c00480_si_001.pdf]

Supporting information

Surface defects and crystal growth of apremilast benzoic acid cocrystals

Jan Jirát[a,b], Vít Zvoníček[a,b], Luděk Ridvan[b], Miroslav Šoóš[a]

[a] *Department of Chemical Engineering, University of Chemistry and Technology,*

Technická 3, 166 28 Prague 6 – Dejvice, Czech Republic

[b] *Zentiva, k.s., U kabelovny 130, 10237, Prague 10, Czech Republic*

Additional data that support information in the manuscript are presented below.

Raman spectra and XRPD patterns were measured and evaluated for the product batches to ensure that desired cocrystal was consistently produced. Measured XRPD pattern was compared with powder pattern obtained from solved structure of the cocrystal.

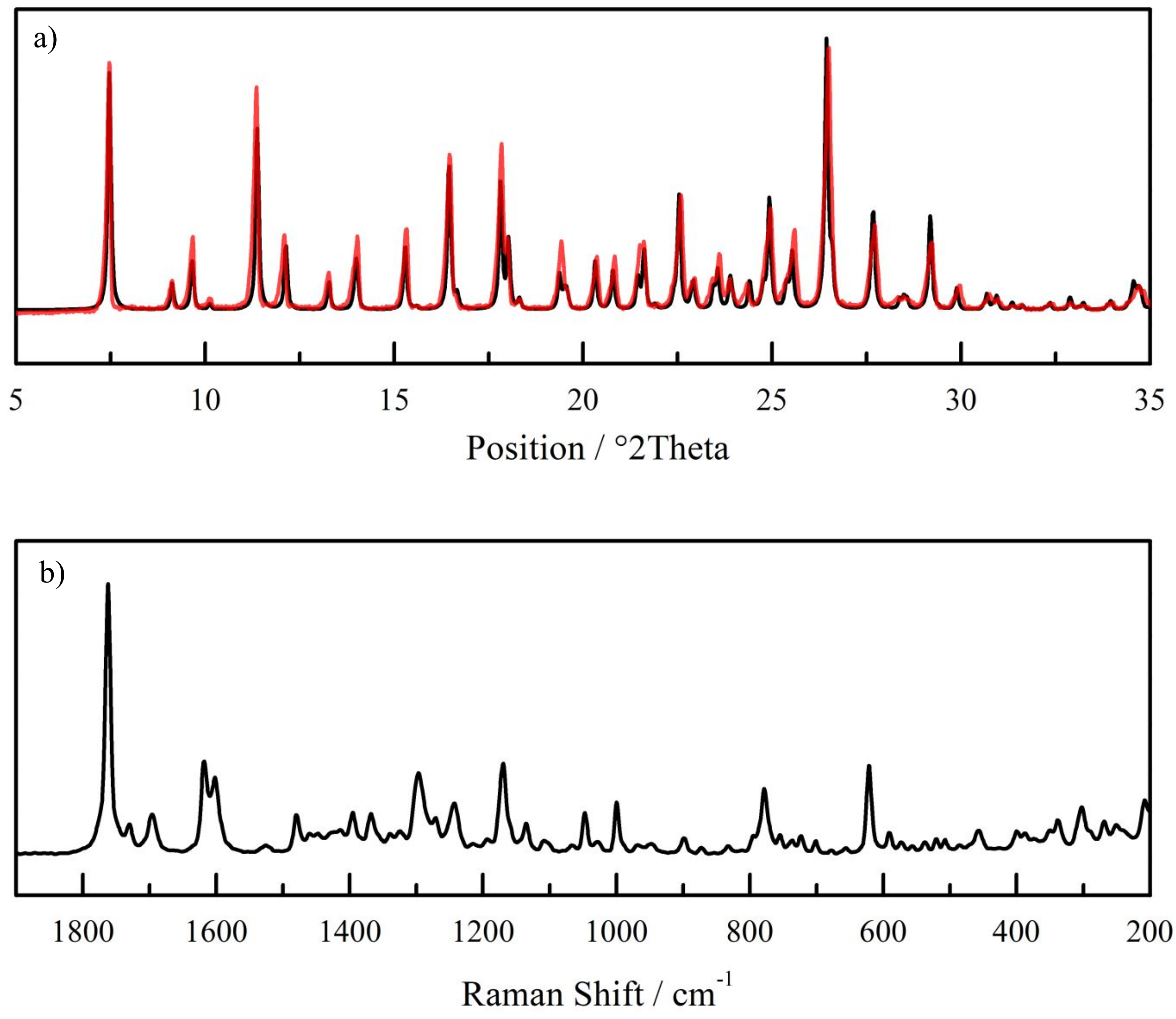

Figure S1: a) comparison of measured and calculated XRPD pattern. Black line – calculated pattern, red line – measured pattern. b) Raman spectra of the cocrystal

The comparison of measured and calculated XRPD pattern confirms formation of the cocrystal in performed experiments. Please note that temperature correction was used in case of the measured XRPD pattern since it was measured at room temperature while the single-crystal measurement was performed at 90 K.

Different cooling rates were explored in terms of crystallization onset temperature. Cooling rates of 0.1 and 0.5 °C/min were displayed in the manuscript. In Figure S2 is plotted faster cooling rate of 2 °C/min to compare with the slower cooling rates.

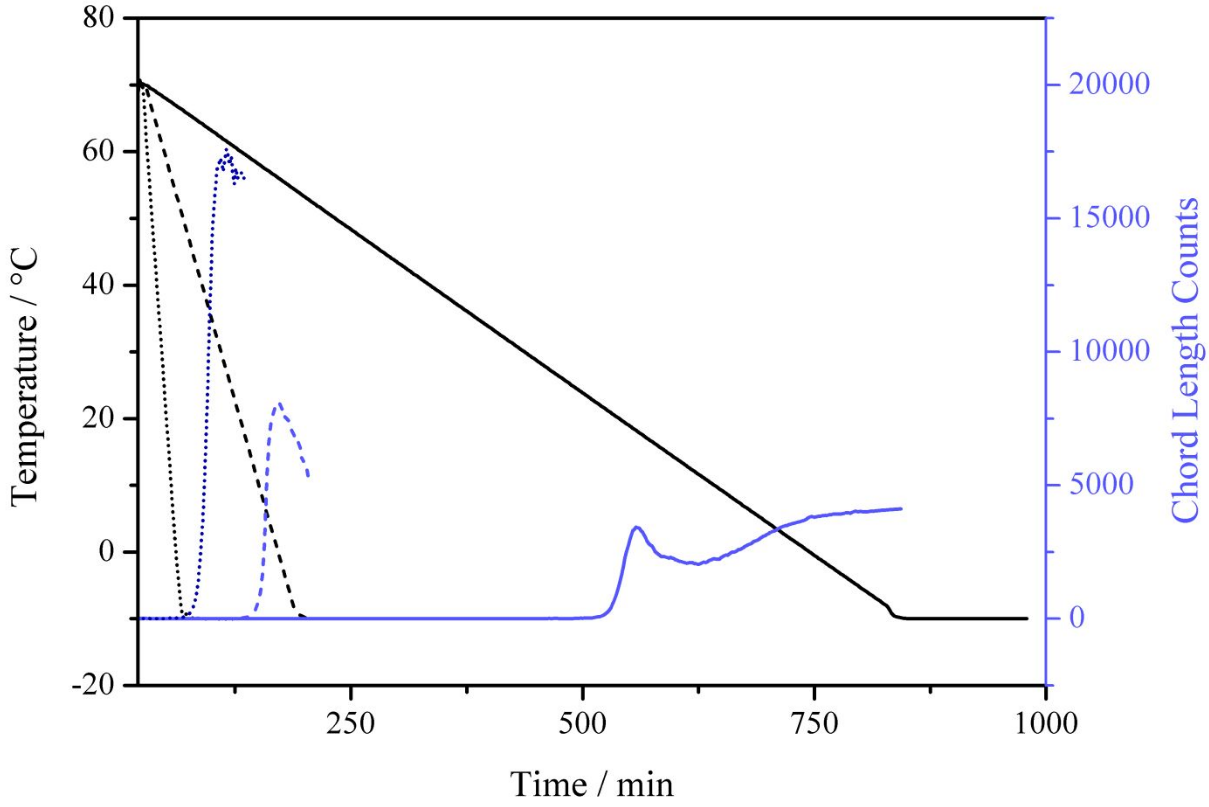

Figure S2: Three cooling rates and chord length count for all experiments, dotted line – 2 °C/min, dashed line – 0.5 °C/min, solid line – 0.1 °C/min. Black line – temperature profile, blue line – chord length count.

The temperature of crystallization onset is approx. 24 °C for the 0.1 °C/min cooling rate, 18 °C for the 0.5 °C/min cooling rate and 5 °C for 2 °C/min. The trend of lower crystallization temperature onset with faster cooling rates is observed throughout all experiments. The mixing time for the fastest cooling rate of 2 °C/min is the shortest of all three experiments. Additional experiment with cooling rate of 3 °C/min was performed as well and the crystallization started after the solution was cooled to the final temperature of -10 °C.

The decrease in the chord count near the end of each experiment is due to settling of the biggest particles at the bottom of the reactor. These particles are not measured by the FBRM probe

which results in overall decrease in chord counts. More intensive mixing was not used to prevent frequent and impactful collisions between crystals.

Optical probe EasyViewer was used alongside the FBRM probe in EasyMax system to obtain further information about the course of the experiments. Optical data for all three experiments are provided in Figure S3.

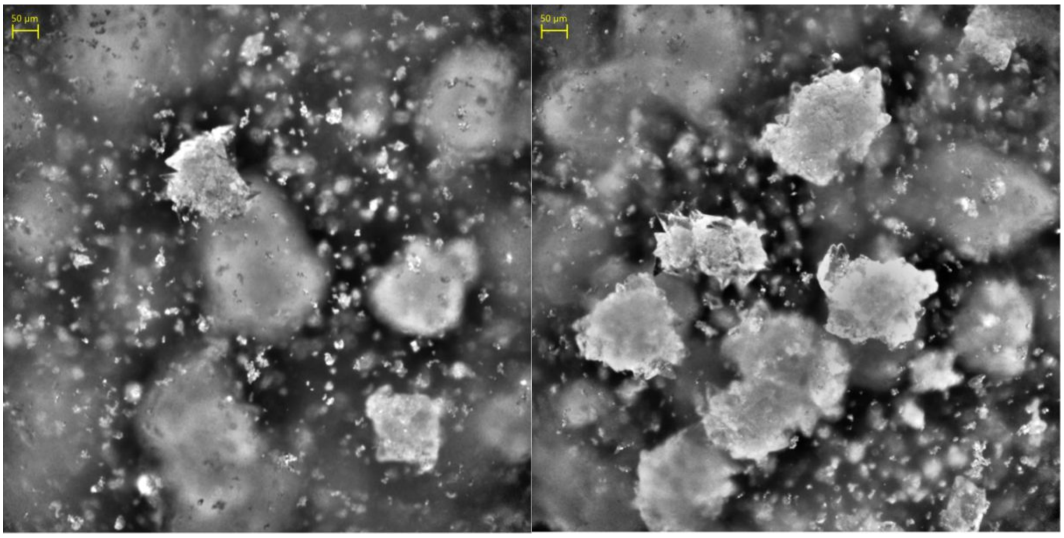

(a)

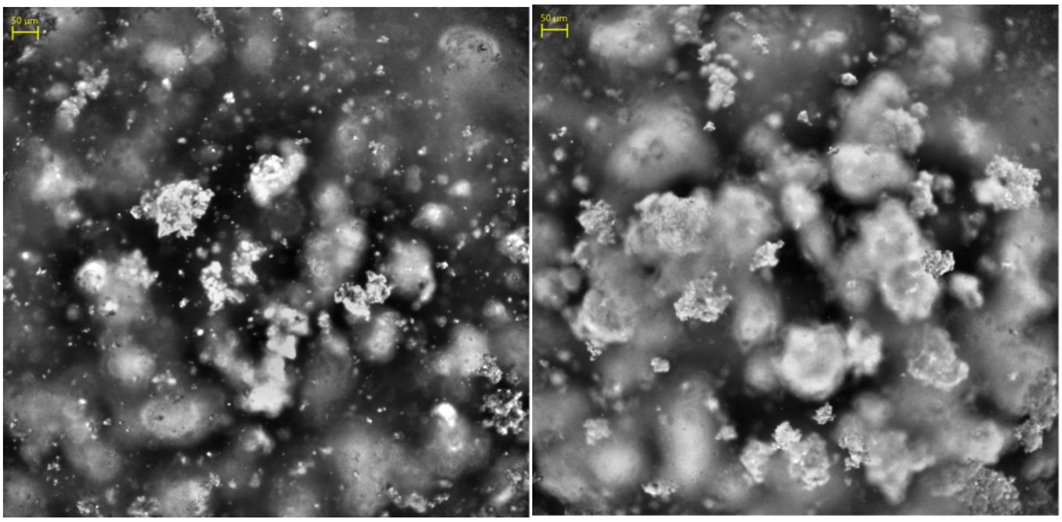

(b)

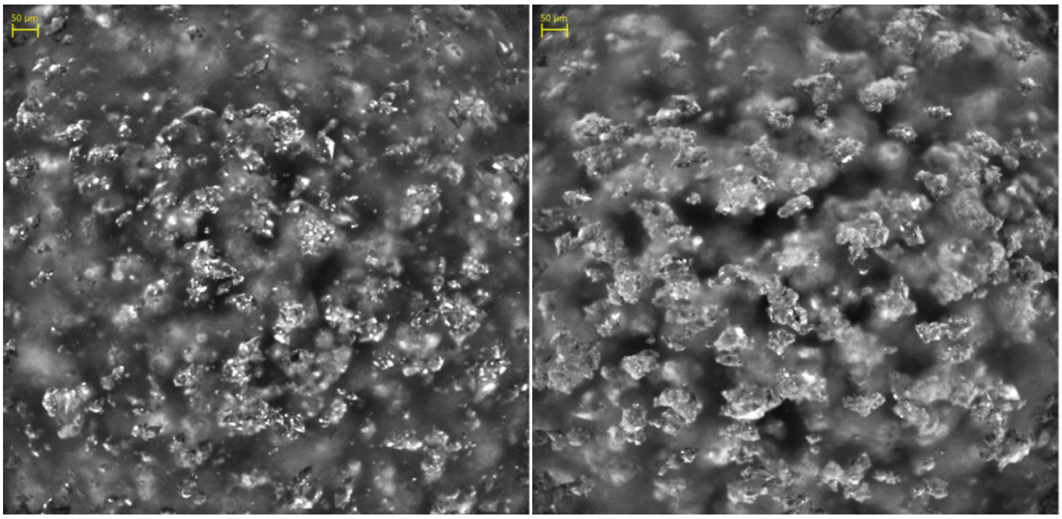

(c)

Figure S3: Optical images of crystals formed in experiments with different cooling rates. a) 0.1 °C/min, b) 0.5 °C/min, c) 2°C/min. Scale bar: 50 μm.

Crystals produced at faster cooling rates are smaller and noticeably more transparent. At faster cooling rates, the crystallization starts at lower temperature, therefore higher supersaturation which results in more nuclei and overall smaller crystals. The mixing time is shorter for faster cooling rates and indents form much later in the experiment. The second growth phase of the crystals is therefore much shorter. This results in non-clustered product that is significantly more transparent.

The duration of the experiment with slowest cooling rate is approx. 15 hours. Small fragments of the crystals form by collisions of crystals over the course of the long experiment.

It is important to note that while using the crystallization system Crystalline (described in the manuscript), separate crystals formed at cooling rate of 0.5 °C/min. For the case of crystallization system EasyMax, separate crystals formed at cooling rate of 2 °C/min. For cooling rate of 0.5 °C/min the product was closer to crystal cluster habit compared to separate crystals. This is due to different hydrodynamic conditions in the crystallization vessel. The size of the vessel, stirrer type, stirrer size all substantially change intensity of mixing and subsequently the frequency and intensity of crystal collisions. Importantly, the key trend of separate crystal formation at faster cooling rate is consistent in both crystallization systems.

For completeness, in Figure S4 in presented comparison of the chord length distributions obtained at the end of the crystallization process for cooling rates 0.1, 0.5 and 2 °C/min. As can be seen, with increasing the cooling rate there is reduction of the crystal sizes. In addition, for slowest cooling rate 0.1 °C/min, there is observed left-tailing of the CLD indicating presence of crystal fragments obtained after attrition of the smaller crystal particles growing on the surface of indented mother crystals as visible in Figure S3.

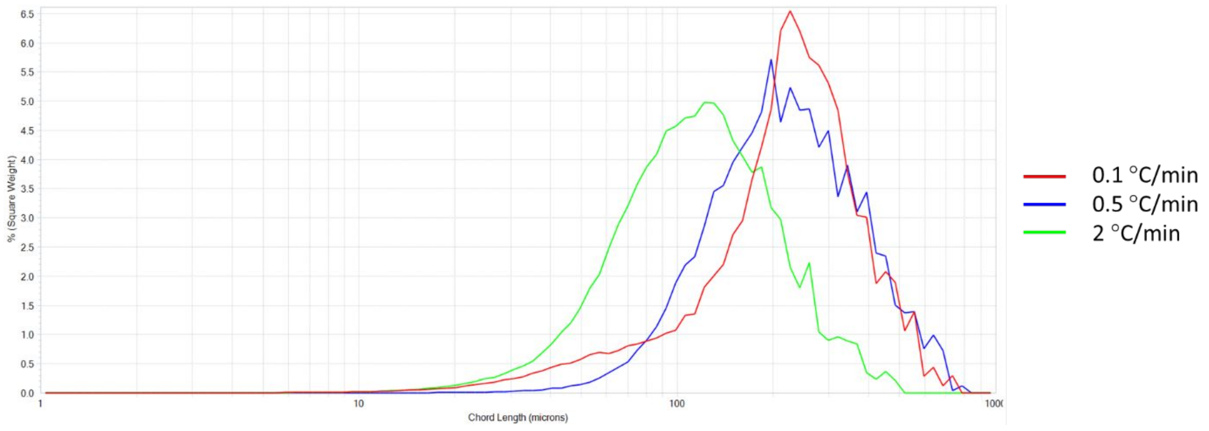

Figure S4: Chord length distribution of prepared crystals measured for 3 various cooling rates

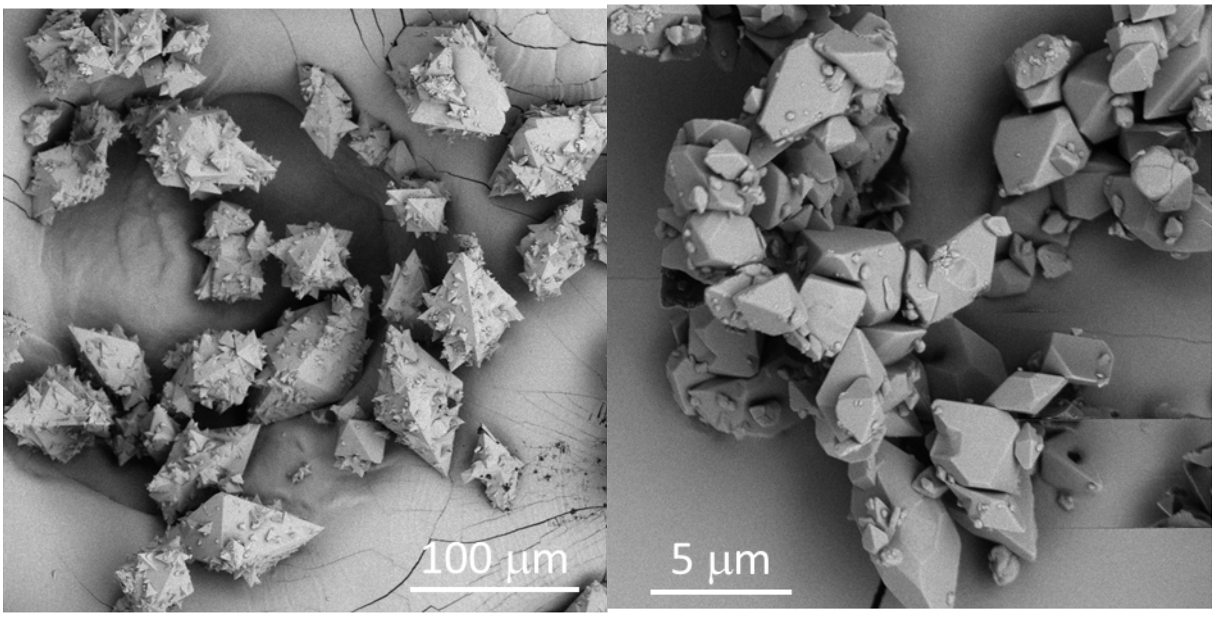

Big bipyramids Small bipyramids

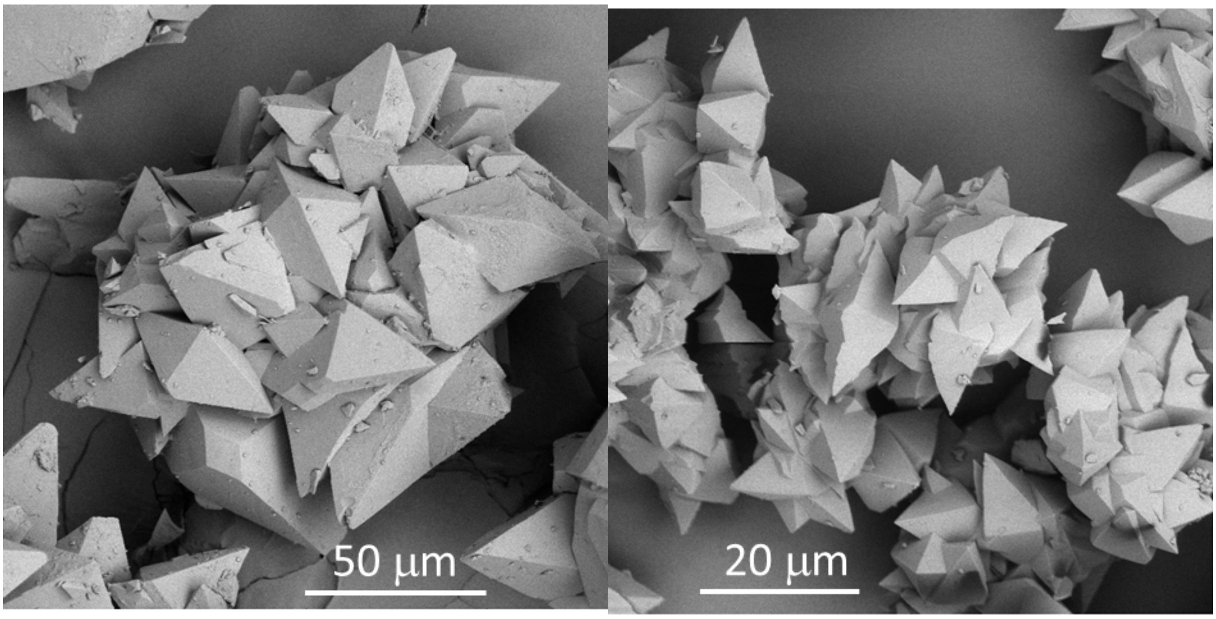

Big clusters Small clusters

Figure S5: Examples of crystals used for dissolution experiment
